# Mass Spectrometry-Based Proteomics of Fungal Pathogenesis, Host–Fungal Interactions, and Antifungal Development

**DOI:** 10.3390/jof5020052

**Published:** 2019-06-17

**Authors:** Brianna Ball, Arianne Bermas, Duncan Carruthers-Lay, Jennifer Geddes-McAlister

**Affiliations:** Department of Molecular and Cellular Biology, University of Guelph, Guelph, ON N1H 1N8, Canada; ballb@uoguelph.ca (B.B.); abermas@uoguelph.ca (A.B.); dcarru01@uoguelph.ca (D.C.-L.)

**Keywords:** fungal pathogenesis, mass spectrometry-based proteomics, host–pathogen interactions, antifungals

## Abstract

The prevalence of fungal diseases is increasing on a global scale, ranging from acute to systemic infections caused by commensal or pathogenic microorganisms, often associated with the immune status of the host. Morbidity and mortality rates remain high and our ability to treat fungal infections is challenged by a limited arsenal of antifungal agents and the emergence of drug resistant pathogens. There is a high demand for new approaches to elucidate the fungal mechanisms of pathogenesis and the interplay between host and pathogen to discover novel treatment options. Moreover, the need for improved drug efficacy and reduced host toxicity requires the identification and characterization of antifungal biological targets and molecular mechanisms of action. Mass spectrometry (MS)-based proteomics is a rapidly advancing field capable of addressing these priorities by providing comprehensive information on the dynamics of cellular processes, modifications, and interactions. In this Review, we focus on applications of MS-based proteomics in a diverse array of fungal pathogens and host systems to define and distinguish the molecular details of fungal pathogenesis and host–fungal interactions. We also explore the emerging role of MS-based proteomics in the discovery and development of novel antifungal therapies and provide insight into the future of MS-based proteomics in fungal biology.

## 1. Introduction

Fungal pathogens cause a diverse array of human diseases, ranging from acute to systemic infections. The onset of infection, whether from a commensal or pathogenic organism, often corresponds with a shift in the host immune status. For example, *Candida* species are commensal to the oral and gastrointestinal tracts in healthy individuals; however, if an individual becomes immunocompromised, the fungi behave as opportunistic pathogens causing invasive candidiasis and candidemia [1,2,3]. Similar trends in fungal pathogenesis occur for *Cryptococcus neoformans* [4] and *Aspergillus fumigatus* [5], whereas infection by *Histoplasma capsulatum* [6] and *Cryptococcus gattii* [7] also occurs in immunocompetent hosts. Although, the source (e.g., natural environment, hospital-acquired) and mode of infection (e.g., inhalation, physical contact), as well as disease symptoms (e.g., fever, hemoptysis, skin lesions, meningitis), vary among fungal species, the morbidity and mortality rates associated with invasion are staggering. For invasive aspergillosis, an important opportunistic infection for neutropenic patients caused by *A. fumigatus*, mortality rates range from 60–90% [8]. Effective treatment strategies to combat fungal infections are limited given the close evolutionary relationship between fungi and the human host. Similarly, the emergence of new, drug-resistant fungal pathogens (e.g., *Candida auris*) represents a significant challenge to current treatment regimens [9,10]. Therefore, novel approaches to elucidate the mechanisms of pathogenesis of fungal species may lead to the identification of new potential targets for therapeutic intervention. In the same light, the exploration of combination therapy and drug repurposing represent innovative strategies for eradicating fungal infections.

**Figure 1:**
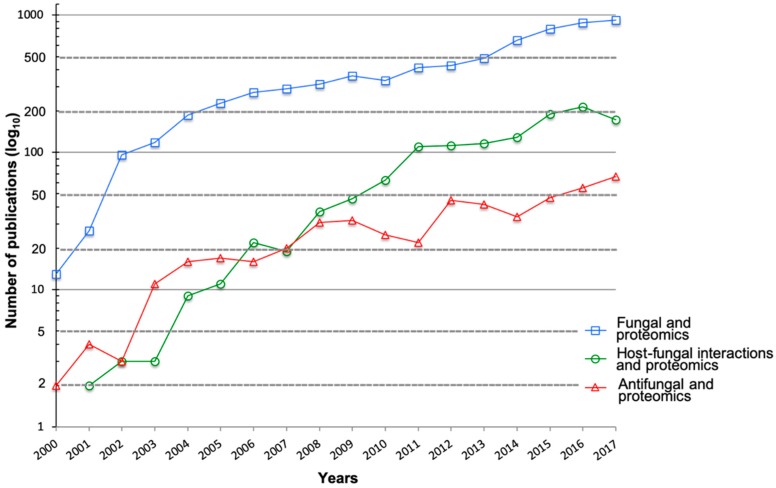
Publications from 2000–2017 of proteomic applications in fungal biology. An in-house developed R-script was used to search PubMed for publications using the following search terms within the abstract and/or title: (i) “fungal OR fungi AND proteomics NOT host”; (ii) “host–fungal interactions OR host–fungi interactions AND proteomics”; and (iii) “antifungal AND proteomics”. Note: y-axis plotted on log_10_ scale for improved data visualization.

Over the past two decades, the field of microbiology has benefited substantially from technological advances in mass spectrometry (MS)-based proteomics. Specifically, the application of MS-based proteomics in the fields of fungal biology, host–fungal interactions, and antifungal development has resulted in a steady increase in contributions, as demonstrated in a search of PubMed publications (Figure 1). Proteomics based on high-resolution MS is a powerful tool for profiling and quantifying proteins within cells, organs, or tissues [11,12]. It provides comprehensive information on the dynamics of cellular processes, modifications, and interactions. Classically defined, discovery-driven proteomics refers to bottom-up or shotgun proteomics, which relies on enzymatic digestion of proteins prior to identification on a mass spectrometer. Conversely, top-down proteomics encompasses the analysis of intact proteins and identification of proteoforms, whereas targeted proteomics focuses on a limited set of predefined peptides in a complex mixture and is often associated with biomarker development.

**Figure 2:**
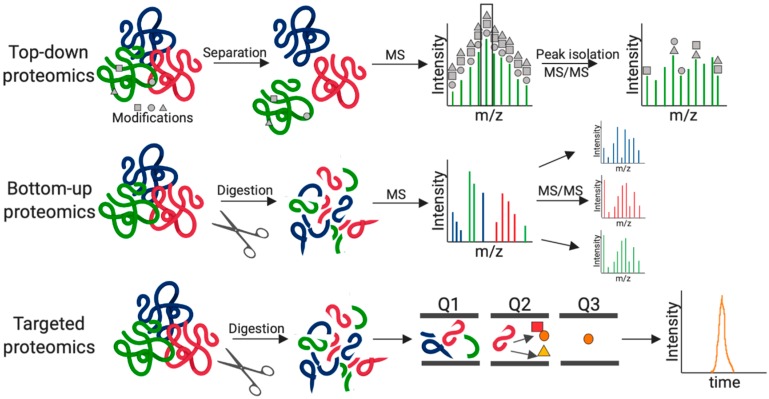
Graphical representation of MS-based proteomics workflows. Top-down proteomics analyzes intact proteins by LC–MS/MS for the identification and quantification of unique proteoforms following separation of proteins by size and peak isolation. Bottom-up proteomics measures proteolytic digested proteins (peptides) by LC–MS/MS for unbiased identification and quantification of proteins within a sample. Targeted proteomics measures a pre-defined set of peptides (isolation of parent ion by mass in Q1, collision of the ion Q2, and mass filtering of product ion in Q3) by LC–MS/MS for identification, characterization, and quantification of specific proteins and biomarkers. Figure generated using BioRender.com.

The general principles of these three MS-based proteomics techniques are outlined in Figure 2. MS analysis begins with sample preparation, which may range from simple solubilization and denaturation of proteins using a chaotropic agent (e.g., urea) to extensive tissue disruption (e.g., probe sonication) and boiling in a detergent (e.g., sodium dodecyl sulfate). For bottom-up and targeted analyses, proteins are digested into peptides using sequence-specific proteases (e.g., trypsin or Lys-C) and purified on C18 resin [13], prior to MS. Notably, for the absolute and relative quantification of proteins or peptides, metabolic (e.g., isotopically stable amino acid incorporation at the cellular level [14,15]), chemical (e.g., addition of mass tags or chemical derivatization [16,17,18]), or label-free [19] approaches are applied. In addition, samples may undergo separation based on size, mass, or charge to reduce sample complexity and promote deeper coverage of the proteome. Next, the first MS scan (MS1) records masses present at a given time and the subsequent MS2 or MS/MS scan selects and fragments peptides or ions for identification based on fragment masses. In traditionally data-dependent acquisition (DDA), the top N most abundant ions are selected for fragmentation; conversely, in data-independent acquisition (DIA) all ions within a defined mass-to-charge (m/z) window are fragmented together and the window is rapidly moved over the entire m/z range. Finally, the processing of modern MS data requires sophisticated bioinformatic workflows and platforms, including MaxQuant, OpenSWATH, DIA-Umpire [20,21,22,23] for analysis, visualization, and interpretation. Presently, there is a movement towards multi-OMICs data integration strategies, for example the combination of genomics, transcriptomics, metabolomics, and proteomics datasets to provide a comprehensive view of a biological system; the connections among OMICs platforms encourages the development of machine learning tools (e.g., Big Data Analytics) and deposition of data into public repositories (e.g., The ProteomeXchange Consortium, PRIDE).

In this Review, we explore the diverse applications of MS-based proteomics to define and characterize the molecular details of fungal pathogenesis and host–fungal interactions over the past five years. Moreover, we present the emerging role of MS-based proteomics in the discovery and development of novel antifungal therapies and provide a perspective on the benefits of adopting proteomics in fungal biology.

## 2. Fungal Pathogenesis by MS-Based Proteomics

**Figure 3:**
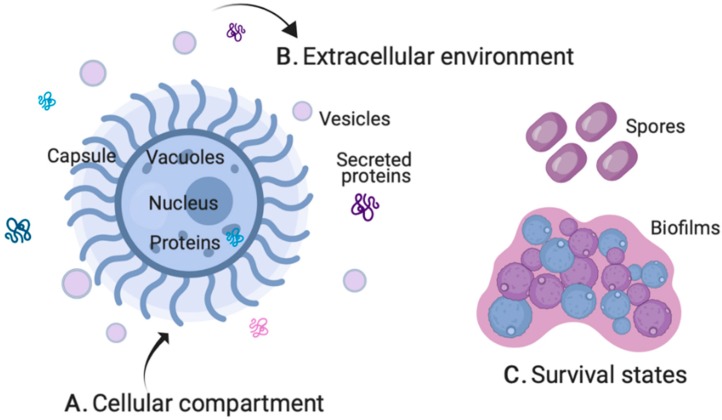
Quantitative proteomics of distinct fungal systems. (**A**) The cellular compartment for total proteome profiling encompasses organelles, the cell wall, intracellular proteins, and polysaccharide capsule as demonstrated in *C. neoformans*. (**B**) The extracellular environment for secretome profiling includes secreted proteins and peptides, as well as vesicles actively or passively released by the cell. (**C**) Survival states include the production of spores or desiccated yeast cells and the formation of biofilms. Figures not drawn to scale; generated using BioRender.com.

Elucidating the mechanisms of fungal pathogenesis by MS-based proteomics enables comprehensive profiling in consideration of molecular structures, modifications, and interactions. The molecular structures may include the cellular compartment (i.e., total proteome), extracellular environment (i.e., secretome and vesicles), and altered microbial states for survival (i.e., biofilms and spores) (Figure 3). Post-translational modifications (PTMs), including some of the most common forms—phosphorylation, glycosylation, and ubiquitination—refer to the covalent and enzymatic modification of proteins following protein biosynthesis, whereas protein–protein interactions (PPIs) include high specificity physical contacts between two or more proteins with roles in predicting protein function and druggability.

### 2.1. Cellular Compartment

The investigation of intracellular proteins provides an overview of cellular regulation processes and signal transduction pathway modulation and suggests connections between protein production and pathogenesis. In the opportunistic pathogen, *A. fumigatus*, label-free quantification (LFQ) proteomics [19] demonstrated the impact of abrogating ergothioneine biosynthesis, a trimethylated and sulfurized histidine derivative that exhibits antioxidant properties, on the cellular proteome [24]. Here, the deletion of *egtA* reduced resistance to elevated H_2_O_2_ and menadione, impaired gliotoxin production, and attenuated conidiation. The proteomic profiling of the wild-type and ∆*egtA* strains under basal vs. reactive oxygen species conditions identified 290 dysregulated proteins, including reductases, oxidases, and stress response proteins and enzymes. These results suggest ergothioneine as an auxiliary antioxidant, required for growth at elevated oxidative stress conditions, and revealed a connection between redox homeostasis, secondary metabolism, and metal ion homeostasis. In *C. albicans*, a comparative analysis of total proteome changes between cells grown under normal conditions (e.g., yeast potato dextrose (YPD) media) and those experiencing high osmolarity salt stress identified a subset of significantly increased proteins focused on central carbon metabolism pathways (e.g., arabitol synthesis and glycerol production) [25]. Further analysis of this protein subset suggested a high degree of specificity for osmotic stress response for osmolyte accumulation during cellular adaptation to stress. This study demonstrates the impact of osmoregulation on proteins and metabolic pathways and highlights the importance of fungal adaptation to changing environmental conditions. Proteomics can also define transitional changes between growth states of fungal pathogens, including the thermodimorphic fungi, *Paracoccidioides* [26]. Here, a large-scale quantitative proteomics analysis on i) mycelium, ii) mycelium-to-yeast transition, and iii) yeast cells revealed metabolic pathway reprogramming and the induction of virulence factors and heat shock proteins during the yeast phase. These results demonstrate the connection between protein production and pathogenesis specific to microbial growth and transition phases.

Fungi also represent valuable microbial systems for MS-based proteomics method development. This was recently demonstrated by the application of improved stable isotope labeling in amino acid cell culture (SILAC) [14] in *C. albicans* [27]. SILAC represents a metabolic labeling strategy involving the incorporation of light- or heavy-labeled lysine and arginine for the accurate quantification of proteins in a sample. Namely, cells are grown in the presence of (i) natural amino acids (light), (ii) ^2^H_4_-lysine and ^13^C_6_ arginine (medium), and (iii) ^15^N_2_^[13]^C_6_-lysine and ^15^N_4_^[13]^C_6_-arginine (heavy) followed by mixing of the differentially labeled samples and analysis together by LC–MS/MS. The mass changes of light-, medium-, and heavy-labeled peptides are detected by the mass spectrometer and allow for the assignment and quantification of a peptide in a specific sample. Traditionally, SILAC relies on auxotrophic mutants for efficient labeling; however, the recent development of Native SILAC (nSILAC) provides efficient labeling during exponential cell growth in the absence of auxotrophic strains [28]. Notably, in *C. albicans*, the use of heavy arginine leads to problems in peak identification due to the occurrence of heavy proline conversion. To overcome this challenge, the authors developed a computational method to correct the protein SILAC (heavy/light) ratios, which normalized the ratios of proline-containing peptides to those of proline-free peptides. Overall, this study provides a strategy for the accurate and quantitative profiling of total proteome changes in *C. albicans* using an nSILAC approach, which has potential applications in other fungal systems faced with similar limitations.

### 2.2. Extracellular Environment

In *C. neoformans*, the cyclic-AMP/protein kinase A (PKA) pathway controls the expression of virulence factors and fungal pathogenesis [29]. A bottom-up proteomic analysis of the PKA-regulated secretome of *C. neoformans* identified 61 secreted proteins—five of which demonstrated significant changes in abundance when PKA expression was abolished [30]. These results informed the design of a targeted proteomics experiment based on multiple reaction monitoring (MRM) to detect and quantify the abundance of the five proteins in murine blood, bronchoalveolar lavage fluid, and infected macrophage lysates. Overall, this study combined the power of discovery-based and targeted proteomics experiments to define the role of PKA in regulation of protein secretion in *C. neoformans* and identified the first biomarkers of cryptococcal infection. Another study focusing on the secretome of *C. neoformans* performed a comprehensive activity-based assay to identify and characterize secreted peptidases (associated with fungal survival and virulence) in culture supernatants [31]. This study employed multiplex substrate profiling by MS [32] to identify cleavage events within a defined 228-member library of diverse peptidases. The authors defined the putative substrate preference of three peptidases, including a secreted aspartyl peptidase (required for low pH survival and virulence [31]) and screened inhibitors to identify a potent in vitro antagonist with probable application as an anti-virulence therapeutic.

Vesicles represent another product of the secretory machinery and pathways with a potential influence on fungal pathogenesis. The proteomic profiling of extracellular vesicles compared to vesicle-free supernatant from *C. albicans* identified 75 proteins unique to the vesicular fraction with highly variable biological functions [33]. Of these, 60% contained a signal peptide or glycosylphosphatidylinositol (GPI)-anchor, suggesting classical modes of secretion. Conversely, the remaining 40% lacked a signal peptide and likely use alternative routes for exportation (e.g., vesicular pathways, non-classical secretion, or proteins capable of performing dual or multiple functions depending on cell localization). Notably, a secreted immunogenic protein, Bgl2, was identified in the vesicular and supernatant fractions and the purified protein was assessed as a vaccine candidate for use against invasive candidiasis. Another example of vesicular proteome profiling also emphasizes the extreme variability of biological processes enriched within vesicles [34]. Here, a proteomic analysis of size-enriched vesicular fractions from *C. neoformans* confirmed the presence of previously defined vesicular proteins (25%) and highlighted the identification of new vesicular-associated proteins (75%), including ribosomal and translation-related proteins, which may play a role in fungal pathogenesis and influencing host cell response to invasion.

### 2.3. Survival States

Fungal biofilm formation provides an advantageous environment for microbial growth and survival through heightened resistance to environmental stressors, the host immune system, and therapeutics [35]. In *C. neoformans*, a proteomic comparison between biofilm vs. planktonic cells measured over 2000 proteins, including 131 proteins unique to the biofilm state [36]. An in-depth characterization of the biofilm-specific proteins revealed a shift in metabolic regulation from the tricarboxylic acid cycle towards energy acquisition, an emphasis on proteins associated with oxidative stress, and the identification of two proteases with potential roles in assisting fungal dissemination during infection. This study identified a multitude of proteins specific to biofilm metabolic activity, maintenance, and resistance, revealing opportunities for future drug targets and therapeutics.

Spores are metabolically quiescent, dormant structures, which represent another opportunity for the protection of fungi from environmental stressors. In *A. fumigatus*, conidia, or spore-like particles, represent the infectious propagule responsible for initiating invasive aspergillosis [37]. Recently, a complementary analysis of the conidial surface proteome using hydrogen-fluoride-pyridine treatment, as well as surface-exposed conidial peptide cleavage by trypsin, identified 148 proteins. Of these proteins, 40% contain a signal peptide, predicted transmembrane helix, or GPI-anchored attachment signal, supporting their presence at the interface between the cell surface and extracellular environment [38]. Importantly, RodA (rodlet layer forming protein associated with conidia formation) was the most abundant protein identified, along with a previously uncharacterized protein (termed: conidial cell wall protein A; CcpA). The subsequent evaluation of the novel conidial cell-surface protein, CcpA, demonstrated a role in virulence and conidial innate immune recognition, but was not linked to cell surface structure. In another example, proteomic profiling between spores and vegetative cells (yeast) of *C. neoformans* identified 18 spore-specific proteins [39]. Here, the deletion of the 18 genes of interest, followed by phenotypic assessment, demonstrated a defect in sexual development (involved in spore formation) and germination (involved in initiation of vegetative growth) in the mutant strains compared to wild-type strains. This analysis revealed unexpected connections between spore production and composition in the pathogenic fungi. Taken together, these studies demonstrate protein-level differences of cells in a sporulated state and suggest mechanisms of virulence and immune system regulation by fungal pathogens.

### 2.4. Modifications and Interactions

The investigation of PTMs can identify direct and indirect targets/substrates of proteins and enzymes, as well as interacting partners. For example, whole phosphoproteome analysis by TiO_2_ enrichment of phosphopeptides aimed to identify substrates of calcineurin (a Ca^2+^/calmodulin-activated serine-threonine-specific protein phosphatase involved in regulation of septation) in *A. fumigatus* [40]. Here, an analysis of proteins with altered abundance associated with phosphorylation between the wild-type and calcineurin-deficient (∆*cnaA*) strains identified Kin1 (a protein kinase important for cell wall stress response and antifungal susceptibility) to be a substrate of calcineurin-mediated dephosphorylation. This study provides novel insight into Kin1 regulation by calcineurin, connecting phosphatase activity with proper cell division. Similarly, in *C. neoformans*, phosphoproteomic screens between calcineurin-activated (WT cells exposed to 37 °C) and calcineurin-deficient conditions (∆*cna1* or WT cells exposed to a calcineurin inhibitor, FK506) identified 56 calcineurin-dependent dephosphorylation targets [41]. These targets include the transactivator Crz1, and proteins with roles in stress response, mRNA binding/stability, protein translation, and vesicular trafficking. Characterization of the relationship between calcineurin and Crz1 support a model in which calcineurin governs growth at high temperature, virulence, and sexual reproduction by controlling both DNA- and RNA-binding proteins in transcriptional and post-transcriptional circuits.

An alternative to global PTM profiling involves immune-affinity enrichment using antibodies. For example, in *H. capsulatum*, the enrichment of lysine succinylation sites captured on an anti-succinylysine antibody identified 463 uniquely modified sites, corresponding to 202 proteins with diverse biological functions and cellular localizations [42]. The in-depth bioinformatic characterization of the identified targets highlights protein interaction networks and metabolic pathways impacted by succinylation and a connection with fungal pathogenesis and proliferation. A year earlier, this group also demonstrated the enrichment of lysine acetylation sites in *H. capsulatum* using peptide prefractionation, antibody enrichment, and LC–MS/MS to define six types of acetylation site motifs and suggest preferred substrates of lysine acetylation [43]. Moreover, the authors report a connection between acetylation and succinylation in the fungus and a role for acetylation in virulence factor regulation.

A key objective in studying the role of proteins, including kinases and phosphatases, is to assemble a list of targets, which requires the identification of physically interacting proteins. In *C. albicans*, affinity-purification MS (AP-MS) was combined with SILAC labeling and a substrate-trapping mutant of Cdc14 (a key player involved in orchestrating mitosis and cell division with dual specificity as a proline- and serine-directed phosphatase) to distinguish genuine interactors from proteins that bound non-specifically to the affinity matrix [44]. The proteomic analysis identified 126 proteins that interact with Cdc14—of which, 44% contain a Cdc14 dephosphorylation motif and 80% were classified as novel interactors, including proteins with roles in cell cycle, cytokinesis, and DNA repair. Taken together, this study provides an expanded list of Cdc14 interactors in *C. albicans* and establishes a robust and quantifiable method for identifying *bona fide* partners of kinases and/or phosphatases in a fungal system.

## 3. MS-Based Proteomics of Host–Fungal Interactions

The interaction between host and microbe is critical for the initial control and clearance of an invading microbe and for predicting disease outcome. Defining the opposing roles of these biological systems from a global perspective (i.e., in consideration of both the host and microbial responses) is imperative to uncovering novel strategies to combat infection. Despite the importance of these interactions to microbe and host health, the critical features and mechanisms governing the interplay between these two systems have not been well characterized at the protein level. Here, we explore the ability of MS-based proteomics to define interactions between host and pathogen during infection, identify key proteins or pathways associated with effective host response, and suggest therapeutic targets for novel treatment strategies.

### 3.1. Host Perspective

Profiling the impact of fungal infection from the host perspective can glean insight into the host immune response, specific proteins or pathways involved in combating infection, and suggest potential drug targets to enhance the host defense mechanisms. Recently, a proteomic analysis using isobaric tags for relative and absolute quantification (iTRAQ) of frontal lobe brain tissues from patients suffering from HIV and cryptococcal meningitis was performed to identify host proteins involved in the process of invasion and infection [45]. Briefly, iTRAQ involves the addition of covalently-bound stable isotope-labeled molecules of different masses to the peptides in a sample, which results in a detectable chromatographic shift in the mass spectrometer and subsequent, assignment of peptide identification and quantification values within each labeled sample [17]. In this study, the authors detected 317 host proteins with differential changes in abundance, including proteins associated with the immune response and signal transduction pathways. The immune response proteins included complement factors, major histocompatibility proteins, as well as caveolin 1 and actin (proteins previously shown to be involved in fungal invasion of the brain); the abundance of five major histocompatibility complex (MHC) proteins were validated by immunohistochemistry. This work demonstrates the proteome differences of the brain during co-infection and highlights the amplified production of important host defense mechanisms upon microbial invasion.

For example, proteome and transcriptome profiling of primary human bronchial epithelial cells grown at the air–liquid interface exposed to *A. fumigatus* conidia showed differential abundance of 153 proteins [46]. Moreover, data integration of RNA expression and protein abundance revealed that genes related to cell cycle regulation, apoptosis/autophagy, iron homeostasis, calcium metabolism, complement and coagulation cascades, translation, ER stress, and unfolded protein response are enriched in the early immune response upon fungal interaction. Although, this study integrated multi-OMICs datasets, it is important to note the limitations of correlating transcript levels with protein abundance [47]. For example, in *C. albicans*, a recent study demonstrated an increase in protein abundance correlated to reported increases in transcript levels; however, a reduction in transcripts did not lead to equivalent changes in protein amounts [25]. Correlation between reduced transcript levels and protein abundance is linked to several factors, including the intracellular stability of the protein (e.g., protein turnover rates) and post-translational regulation [48,49]. For human umbilical vein endothelial cells exposed to *A. fumigatus* hyphae, a label-free, MS-based proteomics analysis detected 89 differentially abundant proteins and also showed regulation of pathways associated with immune response, signaling processes, and homeostasis [50].

Building upon the success of the global proteome profiling of the host response to infection, a focus on phosphoproteome regulation provides valuable insight into the importance of host signaling cascades in fungal clearance. For example, a global phosphoproteomic analysis of the host response to cryptococcal infection of macrophages demonstrated differential phosphorylation of proteins in the AMP-activated protein kinase-autophagy initiation complex (AIC) signaling network [51]. Specifically, the phagocytosis of *C. neoformans*, activates the host AIC and upstream regulatory components, LKB1 and AMPKα, which regulate autophagy induction through their kinase activities. Moreover, the study shows that the recruitment of AIC components to *Cryptococcus*-containing vacuoles regulates the intracellular trafficking and replication of the pathogen, demonstrating that AIC regulatory networks may confer susceptibility to infection due to the modulation of phosphosites involved in the autophagy process.

### 3.2. Pathogen Perspective

Defining the interplay between host and pathogen from the pathogen’s perspective can identify novel virulence factors, characterize new modes of action for previously defined fungal proteins, and uncover anti-virulence strategies to combat infection. For example, in *C. neoformans*, an image-based high-throughput screening assay to probe host–fungal interactions identified a protein S-acyltransferase (PFA4; involved in catalyzing lipid modifications of proteins), which influences fungal adherence and phagocytosis in human monocytic cells [52]. Here, biorthogonal palmitoylome-profiling (metabolic labeling of fatty acids with a palmitic acid analog containing an alkyne group), followed by Click chemistry and MS-based proteomics, identified 72 Pfa4-specific host protein substrates. The classification of these substrates showed the enrichment of proteins associated with cell wall processes, cell wall synthesis, membrane trafficking, signal transduction, and transport. Taken together, this study demonstrates that Pfa4 is a major determinant of cryptococcal pathogenesis and highlights the significance of fatty acid modification in regulating fungal morphology, host interactions, and virulence in vivo, suggesting protein palmitoylation as a potential avenue to be targeted for new antifungal therapeutics. Another study characterizing the interaction between *Paracoccidioides lutzii* and macrophages combined affinity chromatography by the biotinylation of macrophage surface molecules with bottom-up proteomics for the identification of 215 fungal proteins at the cell surface [53]. The authors report the in silico classification of this protein subset according to the presence of sites for N- and O-glycosylation, secretion by classical (signal peptides) and non-classical pathways, subcellular localization, and roles in adhesion. Moreover, a serine protease and fructose-1,6-bisphosphate aldolase were selected for further analysis; these proteins demonstrated an increased abundance in *P. lutzii* upon incubation with macrophages, suggesting involvement in the interaction with host cells during the adhesion process, and representing potential targets for anti-virulence strategies. Finally, an exploration of *Malassezia sympodialis*, a commensal fungus in the human skin mycobiome, profiled the interaction between extracellular vesicles (associated with induction of inflammatory cytokine response) and host skin cells [54]. Here, 110 proteins were enriched in the extracellular vesicles compared to yeast cells, including two characterized allergens. Functional experiments demonstrated active binding and internalization of the vesicles into human keratinocytes and monocytes. This work supports a role for *M. sympodialis* extracellular vesicles during interactions with the skin and lays the foundation for proteomic analyses of fungal proteins and their interacting partners within the host cells.

### 3.3. Dual Perspective

Improvements in MS technologies and bioinformatic platforms for integrated data analysis encourages the investigation of host–pathogen interactions in consideration of both opposing sides in a single experiment. For example, a recent study profiled the host (alveolar macrophages) and pathogen (*A. fumigatus*) responses to phagocytosis by isolating conidia-containing phagolysosomes [55]. Here, the authors detected 637 host and 22 fungal proteins of differential abundance in the phagolysosome and differentiated between responses in the presence or absence of melanin pigment in the conidia. To identify organism-specific proteins, the MS data files were searched against the *Mus musculus* (Uniprot) and *A. fumigatus* (Aspergillus Genome Database, AspGD) databases. From the host’s perspective, vATPase-driven phagolysosomal acidification, Rab5 and Wamp8-dependent endocytic trafficking, signaling pathways, as well as the recruitment of the Lamp1 phagolysosomal maturation marker and lysosomal cysteine protease cathepsin Z were impacted by fungal presence. Conversely, from the pathogen’s perspective, differences in melanin influenced proteins associated with catalase, drug response and mitochondrial unfolded protein response elements, and glyceraldehyde-3-phosphate dehydrogenase. Another example of dual proteome profiling investigates the escaping behavior of *C. albicans* from macrophages [56]. Here, a total of 483 *C. albicans* proteins (227 differentially regulated) and 1253 macrophage proteins (five differentially regulated) were identified. The authors compared conventional proteome analysis, which relies on the isolation and separation of cells depending on biological origin (e.g., fungal or mammalian) and the searching of two organism-specific databases, versus their mixed proteome analysis, which does not separate cell types, but instead allows the processing of all proteins in a sample set and bioinformatically distinguishes proteins into either fungal or mammalian during downstream processing. From the pathogen’s perspective, altered proteins were associated with glucose generation, membrane synthesis, stress response, and other unknown functions. From the host’s perspective, differentially abundant proteins were associated with apoptosis and a chaperone. Notably, bioinformatic separation of dual proteomes is an attractive method to profile both the host and pathogen in a single experiment. However, diligence must be exercised to ensure that peptides mapping to proteins in both databases are excluded from further analysis unless extended data processing can distinguish the origin of the dual-mapped proteins. Taken together, these studies provide a wealth of knowledge regarding fungal infection of host innate immune cells and suggest that investigation from dual perspectives provides a comprehensive, and previously unattainable, view of infection.

## 4. MS-Based Proteomics for the Development of Novel Antifungals

In comparison to antibiotics designed to combat bacterial infections, antifungals present the unique challenge of needing to overcome the close evolutionary relationship between eukaryotic fungal cells and the human host. Specifically, antifungal agents must target the eukaryotic fungal cell while ensuring limited damage and cytotoxicity to human cellular function during treatment [9,10]. Currently, four classes of antifungals are routinely used in monotherapy or in combination, including polyenes, azoles, pyrimidine analogs, and echinocandins. Although these classes of antifungal drugs are often active against infection, challenges associated with off-target host toxicity, limited activity, drug–drug interactions, the requirement for prolonged treatment courses, and the emergence of antifungal drug resistance impede drug efficacy and reliability. Today, MS-based quantitative proteomics plays an important role in the discovery and development of new antifungal treatment strategies to combat the spread of fungal infections. These strategies include defining the mechanisms of antifungal resistance, uncovering microbial interactions with antifungal properties, and discovering new opportunities for drug repurposing, vaccine candidates, and antifungal agents.

### 4.1. Antifungal Resistance Mechanisms

To design new antifungals with increased efficacy, we need to first understand the comprehensive effects of current drugs and how resistance to these drugs develops. For example, a quantitative proteomics analysis using iTRAQ labeling of haploid vs. diploid *C. albicans* strains aimed to identify proteins associated with susceptibility to the ‘gold-standard’ antifungal agent, Amphotericin B [57]. The analysis revealed 100 distinctly abundant proteins between the fungal strains, a focus on proteins associated with oxidative stress response, a key mechanism in Amphotericin B cytotoxicity, and identified alkyl hydroperoxide reductase 1 (*ahp1*) as important in antifungal susceptibility. This study concluded that Amphotericin B tolerance is associated with *ahp1* expression through maintenance of the antioxidant capacity of persister cells in biofilms, suggesting a novel mechanism of antifungal resistance in *C. albicans*.

Another approach to define the mechanisms of resistance towards an antifungal is to profile the proteome in response to drug treatment, as demonstrated recently in *Candida glabrata*. In this study, a novel resistance mechanism of Clotrimazole, an azole antifungal, is defined using iTRAQ-MS from membrane-enriched samples [58]. Following the treatment of *C. glabrata* with Clotrimazole, 12 proteins were upregulated, including four multidrug resistance transporters—two of which were previously characterized and linked to imidazole resistance, and two novel targets. The characterization of the novel targets (CgTpo1_2 and CgTpo1_1) by gene deletion demonstrated the increased susceptibility of the mutant strains to a broad spectrum of antifungals, suggesting a diverse role of these transporters in fungal survival during treatment.

### 4.2. Microbial Competition Showcasing Antifungal Properties

Exploring the interactions between microbial species cohabitating in an environment may uncover proteins produced by either microbe to ensure their survival through symbiosis, competition, or predation. This approach fuels the rapidly advancing field of biocontrol agents and, recently, lead to the discovery of the first new class of antibiotics in over 30 years, lugdunin, which is produced by the bacterium *Staphylococcus lugdunesis* in competition with *Staphylococcus aureus*, found within the nose [59]. Alternatively, studying the interaction between bacteria and fungi in the natural environment has demonstrated the role of bacterial chitinase against fungal cell wall chitin as a contributing factor of the anti-pathogenic effect observed between *Bacillus safensis* and *C. neoformans* and *C. albicans* [60,61]. Similarly, investigation between fungal species can provide insight into the potential for new biocontrol agents. For example, the mycoparastic yeast, *Saccharomycopsis schoenii,* kills the emerging multi-drug resistant *C. auris* [62], but descriptions of the molecular mechanisms underscoring this interaction are not well defined. Here, the integration of quantitative live-cell microscopy assays with genomic, transcriptomic, and proteomic approaches identified genes and proteins overproduced by *S. schoenii* during its predation of model prey cells, *Saccharomyces cerevisiae* [63]. The proteomic profiling of the interaction between *S. schoenii* and *S. cerevisiae* demonstrated the enrichment of proteins associated with catabolic processes and regulation of sulfur metabolic processes during starvation conditions, whereas during predation, cell wall-associated proteins were enriched. Further investigation into the interactions of predation suggested that the overexpression of aspartic proteases correlated with predatory activity and general nutrient limitation was the main trigger for predation. Taken together, this work provides a comprehensive and unbiased analysis of the predatory behaviors of *S. schoenii* and suggests *Saccharomycopsis* yeasts as potential biocontrol agents as an alternative approach to the prolonged and overuse of antifungals, which propagate the development of resistant fungal strains.

### 4.3. Drug Repurposing, Vaccine Design, and New Antifungal Development

Drug repurposing strategies represent innovative opportunities to use clinically approved drugs alone, or in combination with other compounds, to improve efficacy and combat infection. This approach has the potential to reduce the required concentration or duration of treatment for antifungals and, thereby, reduce the risk of fungi developing resistance. A novel drug repurposing strategy was recently suggested to combat cryptococcosis, caused by *C. neoformans*, using an FDA-approved anti-cancer drug to interfere with proteostasis [64]. Here, a quantitative proteomics study of *C. neoformans* under regulation of the cAMP/PKA signal transduction pathway highlighted a clustering of proteins associated with translation and the ubiquitin proteasome pathway. Given the connection between the ubiquitin proteasome pathway, PKA activity, and protein degradation in neurodegenerative disorders [65], further investigation of proteasome function with the inhibitor bortezomib revealed an impact on capsule production and virulence. This study lays the foundation for synergistic drug assays combining bortezomib with commonly used antifungals (e.g., fluconazole, amphotericin B) to treat cryptococcal infection.

Alternative approaches to combating the global spread of fungal disease is to design vaccines capable of inducing protective immune responses against infection. For host response, Th1-type CD4+ T cell-mediated immunity is a critical factor as Th1 cytokines stimulate lymphocyte and phagocyte recruitment, as well as delayed type hypersensitivity response [66]. In *C. neoformans*, a combination of proteomics and immunological methods identified four immunogenic cell wall-associated proteins and three cytoplasmic proteins capable of stimulating a Th1-type response, suggesting novel candidates for vaccine design [67]. Another study performed a quantitative proteomic analysis of 13 species of medically relevant fungi with the aim of developing a pan-fungal or broad-spectrum vaccine to protect against infection by multiple fungal species [68]. Here, several cell wall proteins were identified as possible vaccine candidates with high abundance in multiple species of fungi and no homology to human proteins, including 1,3-β-glucanosyltransferases (Gel1-4, Bgt1, and their homologs), Crf1, Ecm22, and EglC; Crf1 and Gel1 were previously detected as promising vaccine candidates, supporting this proteomics approach for candidate identification.

Finally, two newly developed antifungal prototypes, thiosemicarbazide (TSC) and a camphene derivative of TSC (TSC-C), were shown to possess beneficial medical properties, including the inhibition of *P. lutzii* growth [69]; however, the targets of these antifungals have yet to be elucidated. Here, a chemoproteomics approach involving the immobilization of the compounds on resin, followed by incubation with cell extracts, was employed to identify the interaction partners of TSC and TSC-C in *Paracoccidioides brasiliensis* [70]. The integration of multi-OMICs datasets (e.g., transcriptome and proteome) defined many targets of the compounds’ activity, including damage to mitochondrial membranes, triggering of cell cycle arrest, and inhibition of metabolic processes. Taken together, this analysis showed the significant antifungal activity of TSC and TSC-C towards *P. brasiliensis*, with low levels of mammalian cytotoxicity.

## 5. Conclusions

The examples presented in this Review highlight the diverse applications of MS-based proteomics to elucidate the mechanisms of fungal pathogenesis, the interplay between host and pathogen during infection, and the identification of antifungal targets and mechanisms of action for optimized drug design. In general, MS-based proteomics provides innovative opportunities to study disease mechanisms and drug effects due to its inherent multidimensionality in exploring proteome structure and function. Moreover, recent advances in MS techniques continue to improve the resolution and dimensionality of protein interrogation through deeper coverage, higher sensitivity, novel quantification strategies, PTM profiling, and dynamic capturing of PPIs. In addition, advances in bioinformatic platforms capable of integrating datasets (e.g., transcriptomic, metabolomic, proteomic) expand our ability to study fungal biology from a systems perspective. However, the power of enhancing datasets with complementary biological information (e.g., transcript levels and protein abundance) must be closely examined when interpreting possible correlations between molecular levels. Extensive scientific discussions surrounding the multistep processes in gene expression and discrepancies between RNA transcript levels and protein abundance provide a greater understanding of the underlying design principles of gene expression and encourage a holistic view of cellular regulation. Additional considerations or limitations pertaining to the broad application of proteomics profiling in microbiology include limited access to MS technology due to the high costs of ownership, as well as the demand for advanced technical expertise for instrument operation. Beyond the biology, the demand for publicly available data analysis pipelines and data repositories underscores the need for advanced bioinformatic expertise, statistical processing standards, and transparency when analyzing, interpreting, and presenting OMICs data. Taken together, the widespread adoption of MS-based proteomics in fungal biology provides some initial hurdles to the user, but once a robust pipeline is established, the insights gleaned from the comprehensive profiling of fungal proteomes, interactions during infection, and mechanisms of action for antifungals provide an excess of new scientific avenues to pursue. We envision the future of fungal biology to involve the integration of multi-OMICs datasets driven by the plethora of data generated from the proteomic profiling of fungal systems and the improvement of publicly available and user-friendly bioinformatic platforms to enhance this analysis. Finally, our increasing ability to uncover connections between host and pathogen from dual perspectives, either bioinformatically or experimentally, deepens our understanding of fungal biology and may lead to the discovery of new therapeutic strategies to combat fungal infections on a global scale.
